# A Rare Case of Diffuse Nodular Lymphoid Hyperplasia With Rectal Involvement

**DOI:** 10.7759/cureus.24671

**Published:** 2022-05-02

**Authors:** James R Pellegrini, Jose Russe Russe, Jonathan Arias, Shino Prasandhan

**Affiliations:** 1 Internal Medicine, Nassau University Medical Center, East Meadow, USA; 2 Gastroenterology, Nassau University Medical Center, East Meadow, USA

**Keywords:** hematochezia, lymphoid hyperplasia, colonoscopy, rectum, nodular lymphoid hyperplasia

## Abstract

Nodular lymphoid hyperplasia (NLH) is characterized by the growth of multiple discrete small submucosal nodules specifically confined to the lamina propria and superficial submucosa layers of the intestinal wall. Gastric and rectal involvement of NLH is exceedingly rare. To date, few case reports have described diffuse NLH presenting with multiple submucosal lymphomatous polyposis occurring in the rectum.

Our patient is a 39-year-old morbidly obese Hispanic female who presented to the gastroenterology clinic complaining of intermittent hematochezia for the past six months. Colonoscopy showed diffuse nodularity in the sigmoid colon and rectal mucosa, extending 20 cm from the rectal verge. Rectal biopsies revealed moderate active chronic inflammation predominantly of lymphoplasmacytic cells with areas of lymphoid hyperplasia and focal surface ulceration. Immunohistochemistry stains revealed reactive lymphoid hyperplasia (RLH).

The NLH is a risk factor for extraintestinal and intestinal lymphomas. When encountering lymphoid hyperplasias, the possibility of malignancy must always be considered. It is crucial to monitor patients with NLH via capsule endoscopies, small bowel series, and colonoscopy for surveillance of new lesions in light of the potential for malignancy.

## Introduction

The formation of several distinct small submucosal nodules exclusively limited to the lamina propria and superficial submucosa layers of the intestinal wall characterizes nodular lymphoid hyperplasia (NLH). Gastric involvement of NLH is extremely rare [1]. In the gastrointestinal tract, lymphoid hyperplasia is subdivided into focal and diffuse hyperplasia or local and nodular hyperplasia [1]. To date, few case reports have described diffuse NLH presenting with multiple submucosal lymphomatous polyposis [2]. 

When these mucosal lymphoid aggregates in the small and large bowel it is known as diffuse lymphoid hyperplasia, which is common and benign [3]. These lesions have also been known as reactive lymphoid hyperplasia (RLH), where there are benign lesions reacting to inflammatory conditions associated with chronic erosive gastritis, gastric or rectal ulcers [4]. 

This case report was presented as a poster presentation at the American College of Gastroenterology 2021 conference on October 24th, 2021. 

## Case presentation

A 39-year-old morbidly obese Hispanic female was referred by her primary care physician (PCP) for evaluation of intermittent bloody stools for the past six months mixed with the stools and also with wiping. The patient reported straining with defecation and incomplete evacuation of bowel movement with small-caliber stools (Bristol stool scale type 1). She denied any prior colonoscopy workup, tobacco, illicit drug abuse, or heavy alcohol use. There were no known family members with gastrointestinal tract (GI) tract, uterine or breast cancers in the family. Physical exam was found to be unremarkable with rectal exam revealing no blood, anal fissures, or external hemorrhoids. A digital rectal exam was performed and revealed brown stool, no gross blood, and no melena. 

**Figure 1:**
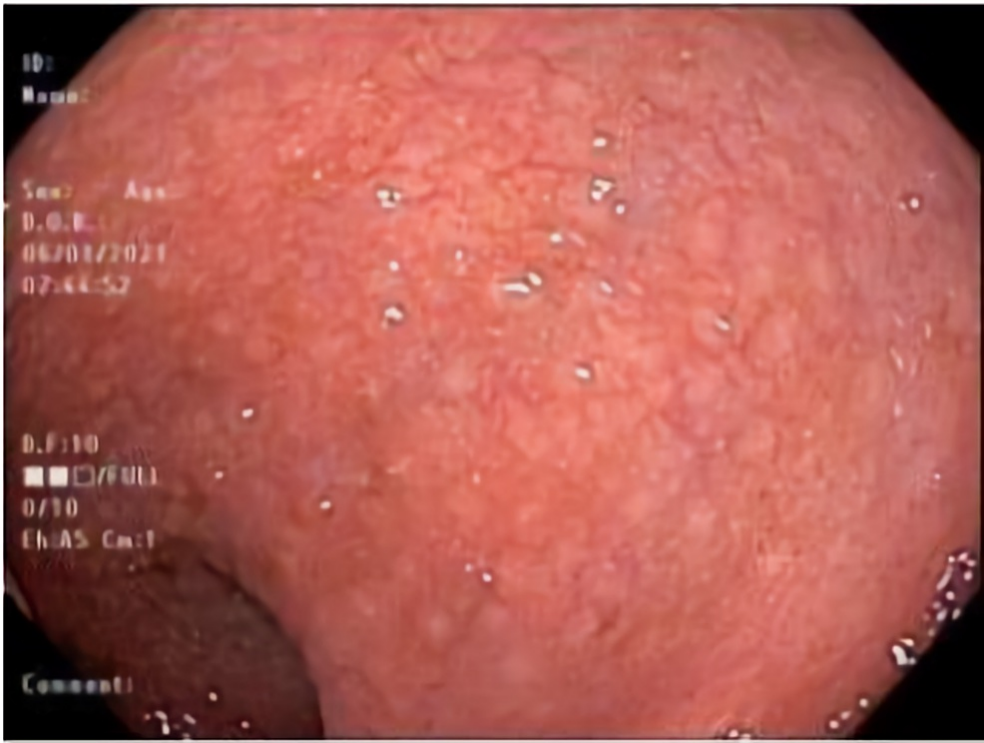
Colonoscopy view of sigmoid colon showing diffuse nodularity.

**Figure 2:**
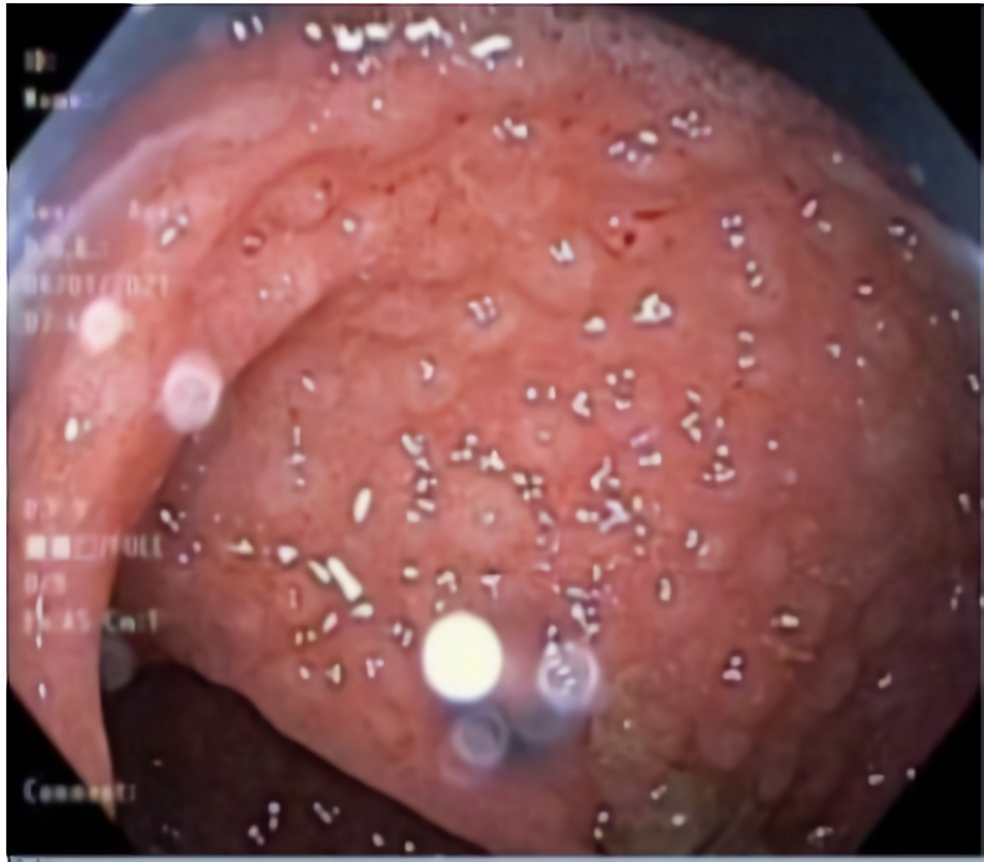
Colonoscopy view of rectum showing diffuse nodularity.

**Figure 3:**
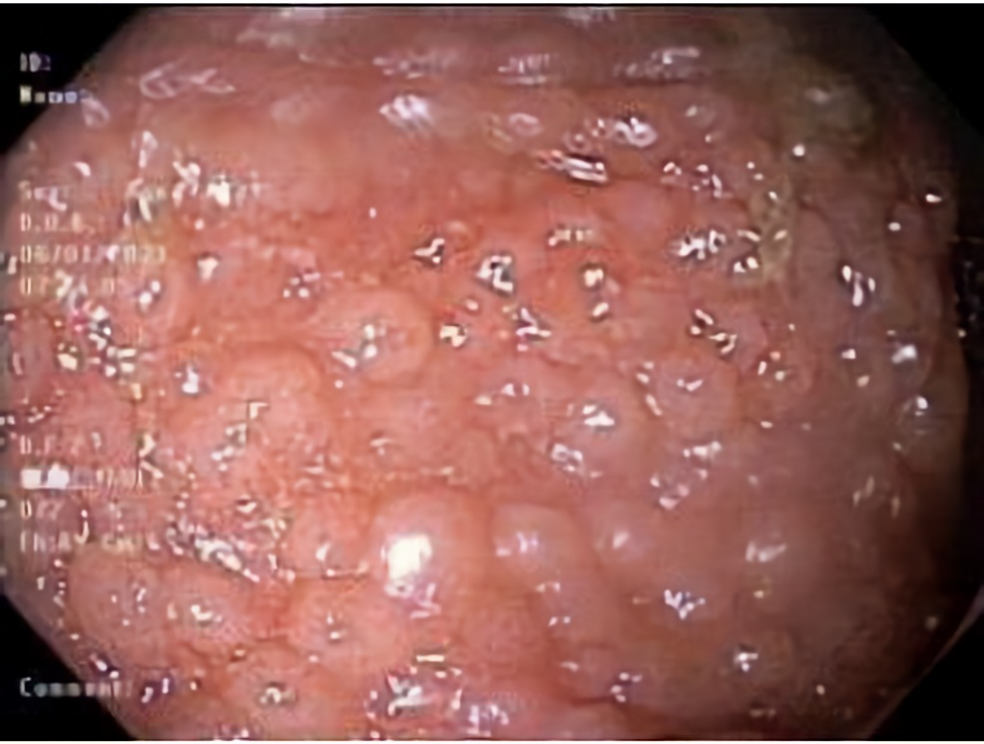
Second colonoscopy view of rectum showing diffuse nodularity.

**Figure 4:**
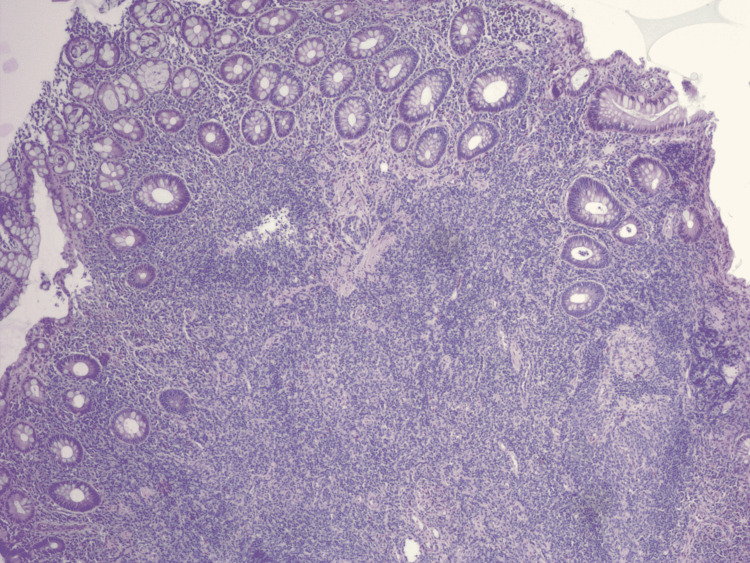
Immunohistochemistry showing large aggregates of lymphoid tissue underlying colonic columnar epithelium with expanded lamina propria.

**Figure 5:**
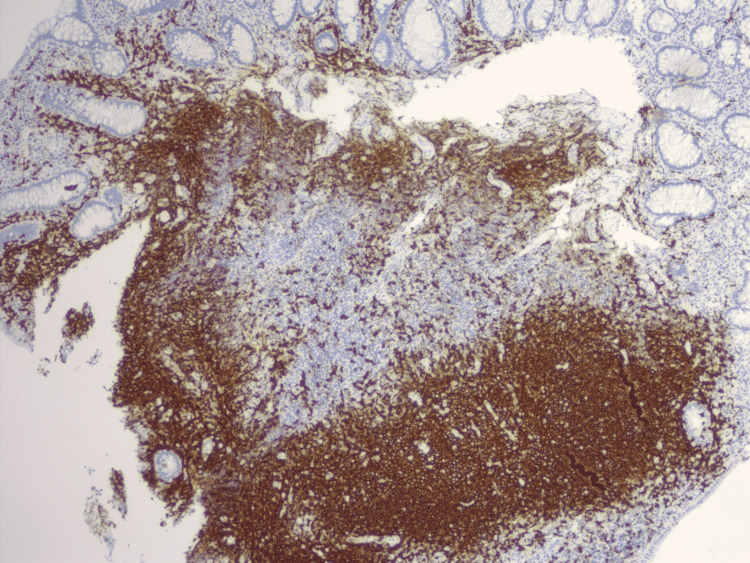
Immunohistochemistry showing CD20 positive-B cells.

**Figure 6:**
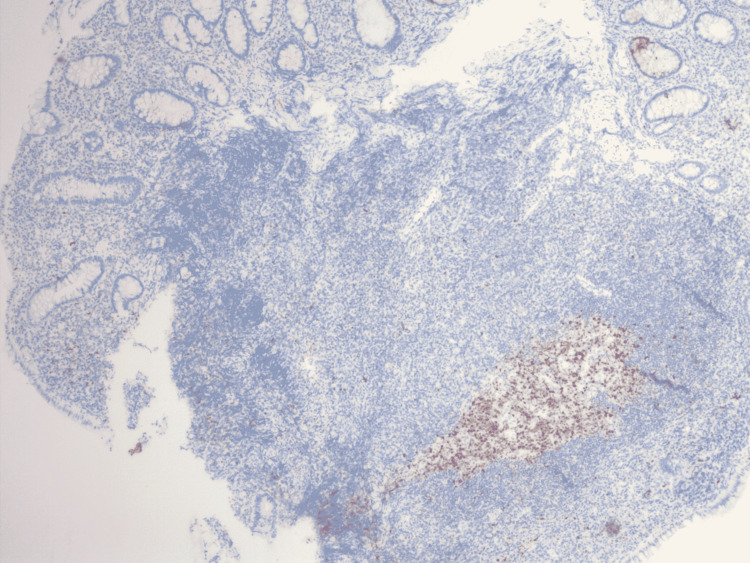
Immunohistochemistry showing bcl-2 positive cells in follicle center.

**Figure 7:**
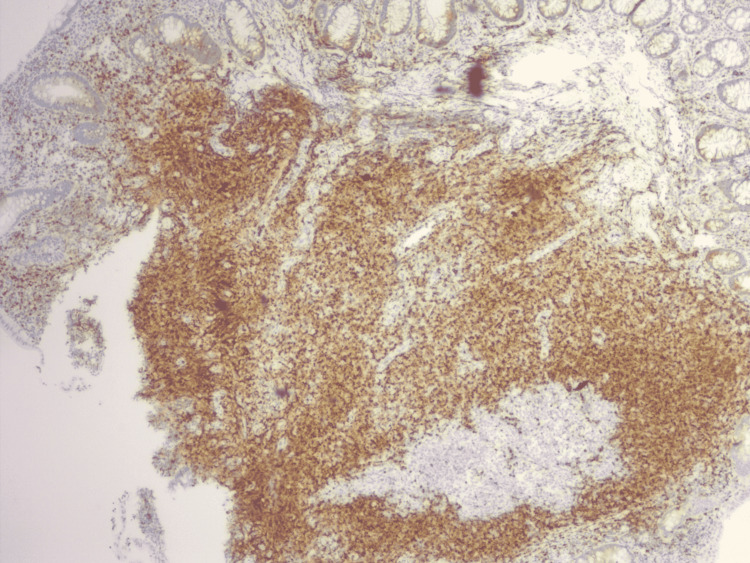
Immunohistochemistry showing CD3 and bcl-2 positive parafollicular T-cells.

The patient was scheduled for a colonoscopy which showed diffuse nodularity in the sigmoid colon (Figure 1) and rectal mucosa (Figures 2-3) extending from the rectal verge to about 20 cm and internal hemorrhoids. Rectal biopsies revealed moderate active chronic inflammation predominantly of lymphoplasmacytic cells with areas of lymphoid hyperplasia and focal surface ulceration (Table 1). Immunohistochemistry stains demonstrated support for RLH (Figures 4-7).

**Table 1:** Rectal biopsy report.

Biopsy number	Report details
Rectal biopsy 1	Moderate active chronic nonspecific inflammation predominantly of lymphoplasmacytic, with patchy areas of lymphoid hyperplasia. No cryptitis or crypt abscesses seen.
Rectal biopsy 2	Mild active chronic inflammation.
Rectal biopsy 3	Moderate active chronic inflammation predominately of lymphoplasmacytic infiltrate with patchy areas of cryptitis and lymphoid hyperplasia. No crypt abscesses seen.
Rectal biopsy 4	Moderate active chronic inflammation predominately of lymphoplasmacytic infiltrate with patchy areas of lymphoid hyperplasia. Focal surface ulceration was also noted. No cryptitis or crypt abscesses are seen.
Rectal biopsy 5	Diffuse active chronic inflammation with markers lymphoplasmacytic infiltrate and large area of lymphoid hyperplasia. No cryptitis or crypt abscesses. Crypt also loss noted.
Rectal biopsy 6	Moderate chronic inflammation with lymphoplasmacytic infiltrates and patchy crypt losses.

The patient was advised to adhere to a high fiber diet, increase oral fluids, and start topical hydrocortisone 2.5% cream for symptomatic relief of her hemorrhoids. It is not known how the patient responded to therapy as she was lost to follow-up.

## Discussion

Hematochezia is a common presentation that encompasses a broad differential. Diffuse NLH with rectal involvement, although uncommon, represents an important differential that must be kept in mind. When encountering lymphoid hyperplasias the possibility of malignant lymphomas always comes to question, however, the actual findings are difficult to classify as benign or malignant [4]. It is distinguished by the polymorphology of the infiltrates, the cytologic atypia, and the reactive lesions within the follicles, however, the definitive diagnosis is mainly given by immunohistochemical or molecular analysis [5].

Lymphoid hyperplasia in the intestine is a rare disorder and diffuse nodular hyperplasia is even more scarce [6]. While the incidence and prevalence are largely unknown, it can occur in patients with or without immunodeficiency. Commonly associated diseases include common variable immunodeficiency, selective immunoglobulin A (IgA) deficiency, Giardia infection, and less commonly in human immunodeficiency virus infection, celiac disease, or *Helicobacter pylori* infection [5]. 

While the pathogenesis for lymphoid hyperplasia is unknown, there are different theories to its cause varying on the presence or absence of associated immunodeficiency disorders. When immunodeficiency is not present, it is believed that NLH is related to immune stimulation of gut lymphoid tissue [5]. An antigenic trigger, such as infection, leads to stimulation and hyperplasia of lymphoid follicles. When immunodeficiency is present, it is proposed that there is an accumulation of plasma-cell precursors within lymphoid tissue [5]. Since NLH is a risk factor for intestinal lymphoma, it has been hypothesized that it can act as a transitional stage for the development of malignant lesions [7].

A study of 781 non-protruding colorectal neoplasias (adenomas and early carcinomas) in Sweden and Japan, suggested an association with colonic lymphoid nodules [8]. NLH is a risk factor for extraintestinal and intestinal lymphomas [7, 9]. The concept of RLH seems to be a spectrum ranging from lesions of completely benign lymphoid hyperplasia to malignant lymphomas, but in many cases, they can be classified as pre-lymphomatous [4]. This is the main reason why surveillance with capsule endoscopies and small bowel series are recommended [5]. 

The focus on the management of lymphoid hyperplasia does not usually require treatment, and is typically directed towards concomitant disorders. Patients with NLH and *H. pylori* infection were studied by Albuquerque et al. In contrast to patients with chronic *H. pylori* infection, those with eradicated *H. pylori* had a substantial clinical response and lesions regression/resolution [5]. However, it has also been reported that patients with NLH and Giardia infection who underwent successful treatment of the infection did not lead to a regression in the number or size of the lymphoid nodules [10]. 

## Conclusions

Diffuse NLH with rectal involvement is a rare disease and differentiating it from other gastrointestinal diseases is a clinical challenge. We report a case of NLH with no notable prior medical history or risk factors. Due to the potential of malignant transformation, periodic surveillance is recommended. Appropriate investigations are needed to better understand the etiology, pathogenesis, and proper management to improve long-term survival in patients.
